# *Syzygium aqueum* (Burm.f.) Alston Prevents Streptozotocin-Induced Pancreatic Beta Cells Damage *via* the TLR-4 Signaling Pathway

**DOI:** 10.3389/fphar.2021.769244

**Published:** 2021-11-29

**Authors:** Mona F. Mahmoud, Shimaa Abdelaal, Heba Osama Mohammed, Assem M. El-Shazly, Rachid Daoud, Mohamed A. O. Abdelfattah, Mansour Sobeh

**Affiliations:** ^1^ Department of Pharmacology and Toxicology, Faculty of Pharmacy, Zagazig University, Zagazig, Egypt; ^2^ Department of Human Anatomy and Embryology, Faculty of Medicine, Zagazig University, Zagazig, Egypt; ^3^ Department of Pharmacognosy, Faculty of Pharmacy, Zagazig University, Zagazig, Egypt; ^4^ African Genome Center, Mohammed VI Polytechnic University, Ben Guerir, Morocco; ^5^ College of Engineering and Technology, American University of the Middle East, Kuwait; ^6^ AgroBioSciences, Mohammed VI Polytechnic University, Ben Guerir, Morocco

**Keywords:** *Syzygium aqueum*, TLR-4, TRAF-6, MyD88, HO-1, diabetes

## Abstract

Although several treatments are available for the treatment of type 2 diabetes mellitus, adverse effects and cost burden impose the search for safe, efficient, and cost-effective alternative herbal remedies. *Syzygium aqueum* (Burm.f.) Alston, a natural anti-inflammatory, antioxidant herb, may suppress diabetes-associated inflammation and pancreatic beta-cell death. Here, we tested the ability of the bioactive leaf extract (SA) to prevent streptozotocin (STZ)-induced oxidative stress and inflammation in pancreatic beta cells in rats and the involvement of the TLR-4 signaling pathway. Non-fasted rats pretreated with 100 or 200 mg kg^−1^ SA 2 days prior to the STZ challenge and for 14 days later had up to 52 and 39% reduction in the glucose levels, respectively, while glibenclamide, the reference standard drug (0.5 mg kg−1), results in 70% reduction. Treatment with SA extract was accompanied by increased insulin secretion, restoration of Langerhans islets morphology, and decreased collagen deposition as demonstrated from ELISA measurement, H and E, and Mallory staining. Both glibenclamide and SA extract significantly decreased levels of TLR-4, MYD88, pro-inflammatory cytokines TNF-α, and TRAF-6 in pancreatic tissue homogenates, which correlated well with minimal pancreatic inflammatory cell infiltration. Pre-treatment with SA or glibenclamide decreased malondialdehyde, a sensitive biomarker of ROS-induced lipid peroxidation, and restored depleted reduced glutathione in the pancreas. Altogether, these data indicate that *S. aqueum* is effective in improving STZ-induced pancreatic damage, which could be beneficial in treating type 2 diabetes mellitus.

## Introduction

Diabetes mellitus is a chronic endocrine metabolic disorder that takes place because of absolute or partially deficient insulin secretory response by pancreatic cells or sometimes due to developed insulin resistance by the body (De Fronzo et al., 1997). Recent statistical estimates showed that around 425 million people are living with diabetes worldwide, with a toll of deaths directly attributed to the disease of approximately 1.6 million per year (Forouhi et al., 2019).

Diabetes involves mainly disrupted carbohydrates metabolism, which translates into impaired glucose usage by body cells resulting in elevated blood sugar level (hyperglycemia). However, metabolism impairment affects lipids and proteins as well (De Fronzo et al., 1997). There are four types of diabetes: insulin-dependent diabetes mellitus (IDDM, type 1), an autoimmune disease characterized by absolute insulin deficiency and affects mainly children and young adults; non-insulin-dependent diabetes mellitus (NIDDM, type 2), also known as adult-onset diabetes and characterized by partial insulin deficiency and/or cells-acquired insulin resistance; gestational diabetes mellitus (GDM), a kind of glucose intolerance during pregnancy; monogenic types of diabetes comprising disorders of genetic defects of beta cells (Alberti and Zimmet 1998). Poorly managed diabetes can cause many complications over the whole body such as renal failure, neuropathy and nerve damage, retinopathy and vision loss, foot ulcers, and infections that might lead to limb amputation, peripheral artery disease, and about 2- to 3-fold increased risk of cardiac attacks and strokes (Harris 1993; Papatheodorou et al., 2018).

Several therapeutic strategies are currently adopted for the proper management of diabetes. Sulphonylureas and meglitinides exert their hypoglycemic effect through stimulating insulin production and increasing its effectiveness in the body. Metformin hinders hepatic gluconeogenesis and modulates lipids metabolism, thus enhancing insulin sensitivity. Thiazolidinediones increase insulin sensitivity by stimulating PPARɤ receptors and the lately developed dipeptidyl peptidase-4 (DPP-4) inhibitors work by elevating incretin levels, hence decreasing glucagon and increasing insulin secretion. However, many adverse reactions are being reported following the usage of some of these therapies, posing higher demand for searching for new alternative targets and safer drugs for proper control of the disease (Tahrani et al., 2016; Padhi et al., 2020). Natural plant-based antioxidants could serve as antidiabetic agents with multiple molecular targets and lower side effects (Kong et al., 2021; Ojo et al., 2021; Shehadeh et al., 2021; Sun et al., 2021).

Several pharmacological activities, including antidiabetic activity, were reported to many plant species belonging to the genus *Syzygium* from the family Myrtaceae (Mahmoud et al., 2021). The species *Syzygium aqueum*, also known as watery rose apple, is native to Malaysia and Indonesia but currently distributed all over the tropics (Sobeh et al., 2016; Mabberley 2017). The plant is widely used in folk medicine. We previously described substantial antioxidant, hepatoprotective, anti-inflammatory, and pain-killing activities from the leaf extract (Sobeh et al., 2018). This wide array of activities was mainly attributed to a plethora of phenolic compounds such as vescalagin, castalagin, epigallocatechin, epigallocatechin gallate, myricitrin, myrilgalone G and B, samarangenins A and B, phloretin, and europetin 3-*O*-rhamnoside (Sobeh et al., 2018).

In the current work, we investigated the antidiabetic potential of a bioactive leaf extract of *S. aqueum* in the STZ diabetes model by exploring the effects of the extract on a wide array of biochemical and molecular markers such as pancreatic malondialdehyde (MDA), reduced glutathione (GSH), tumor necrosis factor-alpha (TNF-α), nuclear factor-erythroid factor 2 (Nrf-2), toll-like receptor 4 (TLR-4), tumor necrosis factor receptor-associated factor (TRAF6), myeloid differentiation factor (MYD88), and heme oxygenase isozyme-1 (HO-1) along with glucose, fructosamine, and insulin levels in plasma. In addition, a detailed histopathological study of pancreatic sections following different treatments was established.

## Material and Methods

### Plant Material and Extraction

*Syzygium aqueum* (Burm.f.) Alston leaves were collected from trees grown in a private garden 30 km from Cairo on Cairo-Alexandria desert road and voucher specimen of the plant material is kept in the Pharmacognosy Department, Faculty of Pharmacy, Ain Shams University (voucher No: PHG-P-SA-181) (Sobeh et al., 2016). The leaves were air-dried, ground, and extracted with 100% methanol at room temperature (100 g, 3 × 500 ml). The combined mixture was filtered and reduced under vacuum at 40°C yielding a semisolid residue that was frozen at −70°C and lyophilized to give a fine dried powder (12 g) (Sobeh et al., 2018).

### Animals and Experimental Design

Male Wistar rats (weighing 200–220 g) were supplied by the animal house of the Faculty of Veterinary Medicine, Zagazig University, Zagazig, Egypt. The rats were kept in polypropylene cages (5/cage) at 25°C, relative humidity of 55%, and a 12 h light/dark cycle. Animals were fed a standard pellet diet with water *ad libitum* and left for a week before the experiments started for acclimation. Thirty rats were divided into five groups (n = 6): normal control, diabetic animals (STZ rats), SA (100 and 200 mg/kg), and reference drug glibenclamide (GLB) (0.5 mg/kg). The extract or glibenclamide was suspended in distilled water containing 5% v/v tween 80 and administered to animals by oral gavage using an orogastric tube. The vehicle (5% v/v tween 80), SA, or GLB was administered 2 days before and for 14 days following STZ injection (Mahmoud et al., 2021).

### Diabetes Induction

Before the experiment, rats were fasted overnight and allowed free access to water. The freshly prepared STZ (50 mg/kg, i. p, citrate buffer, pH 4.5, 0.1 M) was injected (ip) into the overnight fasted rats to induce diabetes. Rats were fed on water containing 5% glucose for 24 h following STZ injection to guard against severe hypoglycemia (Mahmoud et al., 2021).

### Blood and Tissue Sampling

At the end of the experiment, blood samples were collected from the tail vein of the non-fasted rats to determine plasma glucose level using ONE-TOUCH glucometer and test strips from Accu-Chek (Roche Diagnostics GmbH, Mannheim, Germany), with units in mg/dL. Spot urine was also collected to test for glucosuria using urine strips (Teco Diagnostics, CA, United States). Animals were then euthanized by decapitation under anesthesia with ketamine/xylazine (90 mg/kg/9 mg/kg, i. p.) on day 14 of the experiment. The trunk blood was collected, and serum was separated by centrifugation at 3,000 × g for 15 min in a refrigerated centrifuge (High-Speed Refrigerated Centrifuge TGL-16C, China). Serum was kept at −20°C for further biochemical analyses. The pancreas tissues from the rats with different treatments were collected and divided into two parts. The first part was snap-frozen in liquid nitrogen and stored at −80°C for tissue biochemical and molecular analyses and the second part was preserved in a 10% neutral buffered formalin solution for histological examination. The liver was also separated, snap-frozen and stored at −80°C for detection of insulin signaling pathways (IRS-2, P-AKT and GLUT4) (Mahmoud et al., 2021).

### Biochemical Analyses

Glucose, fructosamine, and insulin levels were evaluated according to the previously reported standard procedures. Glucose and fructosamine levels in plasma were measured spectrophotometrically, while the level of serum insulin was assessed using a rat ELISA kit for insulin (Assay Kit Co., Ltd., Sunnyvale, United States) (Mahmoud et al., 2021).

### Assessment of Insulin Receptor Signaling Pathway

The insulin sensitivity, IRS2, P-AKT, and GLUT4 levels were assessed in the liver tissues of the rats by ELISA kits according to the manufacturer’s protocol (Mahmoud et al., 2021).

### Evaluation of Pancreatic Oxidative Stress Markers

Malondialdehyde (MDA) and reduced glutathione (GSH) contents were measured according to the described protocols of Ohkawa et al. (Ohkawa et al., 1979) and Ellman (Ellman 1959). Nuclear factor-erythroid 2 (Nrf2) was measured by ELISA kits in the animals’ pancreatic tissue homogenates according to the manufacturer’s protocol (Lewis et al., 2015).

### Assessment of Inflammatory Markers

TNF-α and TLR-4 were measured in the animals’ pancreatic tissue homogenates by ELISA kits according to the manufacturer’s protocol (Mahmoud et al., 2021).

### Histopathological Studies

Pancreatic tissues were fixed in neutral formalin solution for 48 h. Then, they were dehydrated by passing through a graded sequence of alcohol and embedded in paraffin blocks. Sections were obtained at a thickness of 5 µm using Leica RM 2135 rotary microtome then mounted on glass slides. Sections were deparaffinized in xylene and stained with hematoxylin and eosin stain (HE) and Mallory trichome stain according to Bancroft and Gamble (Bancroft and Gamble 2008; Mahmoud et al., 2021) and then examined using a light microscope (LEICA, ICC50 HD, Switzerland).

### Immunohistochemical Studies

Serial sections of 4 μm thickness each were immersed in 10 mM citrate buffer and heated in a water bath for 30 minutes. Each slide with a mounted section (two sections/slide) was allowed to cool down for 20 min and then washed under running tap water. Sections were then treated with 3% hydrogen peroxide in methanol for 15 min to inactivate the endogenous peroxidase activity. Nondefinite protein binding was blocked using the normal goat serum provided in the Vectastain Universal Elite ABC kit (Vector Laboratories). Then, sections were incubated with primary antibodies against TRAF6 antibody (1:200) and MyD88 (1: 250) (Abcam, United States) overnight at 4 C and anti-HO-1 rabbit polyclonal antibody (Stressgen Biotechnologies, Canada) for 2.5 h and later with HRP (horseradish peroxidase)-conjugated secondary antibodies (Zhongshan Goldernbridge Biotechnology Co., Beijing, China) for 60 min at room temperature. The color was developed using 3,3-diaminobenzidine (DAB) solution. The negative control was obtained by sections incubated in the absence of the primary antibody. The sections were counterstained with 0.1% hematoxylin for 5 min (Shen et al., 2020; Luo et al., 2021).

### Morphometric Analyses

Morphometric analysis procedures were performed at the imaging unit of Anatomy and Embryology Department, Zagazig University, using ImageJ IHC Profiler plugin for ImageJ software. Mallory-stained sections were analyzed to calculate the area% of collagen at a magnification of ×400. Immunostained slides with anti-TRAF6, anti-MYD 88, and anti-HO -1 were captured at ×400 magnification and the percentage of positive expression (areas stained with brown color) was calculated.

### Statistical Analysis

The data were expressed as means ± standard error of mean. The statistical difference among the groups was assessed using the One-way Analysis of Variance (ANOVA), with the Tukey *post hoc* test. The significant variance between the experimental groups was considered at p ˂ 0.05. GraphPad Prism version 8 (GraphPad Software Inc, La Jolla, CA, United States) was utilized to perform the statistical analysis of the data.

## Results

We previously characterized 87 secondary metabolites from SA leaf extract using LC-ESI-MS/MS. These major compounds include myricitrin with several glucosides and galloylated derivatives from the flavonols myricetin and quercetin and epigallocatechin gallate (EGCG) along with several proanthocyanidins, ellagitannins, several dihydrochalcones, such as myrigalone B and G, and their glucosides, in addition to several phenolic acids and their glucosides (Sobeh et al., 2018), Figure 1.

**FIGURE 1:**
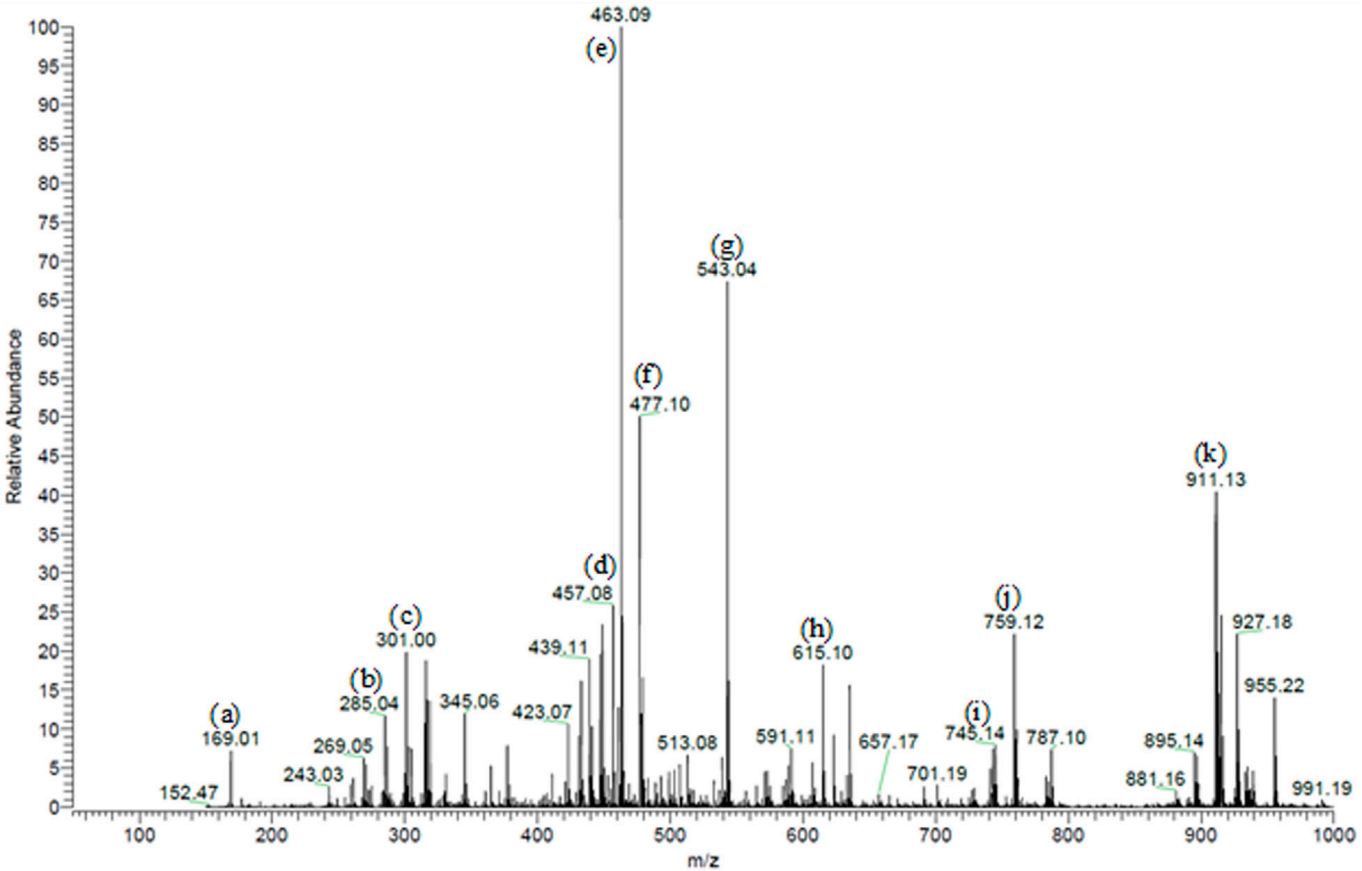
Partial MS profile of *S. aqueum* leaf extract in the negative-ion ESI/MS. **(A)** Gallic acid, **(B)** myrigalone-G, **(C)** ellagic acid, **(D)** EGCG, **(E)** myricitrin, **(F)** europetin 3-rhamnoside, **(G)** myricetin coumaroyl sulphate, **(H)** myricetin galloyl rhamnoside, **(I)** (epi)-gallocatechin-(epi)-catechin gallate **(J)** samarangenin A, and **(K)** samarangenin B.

### Effects of SA on the Levels of Blood Glucose, Serum Fructosamine, and Serum Insulin in STZ Animals

**FIGURE 2:**
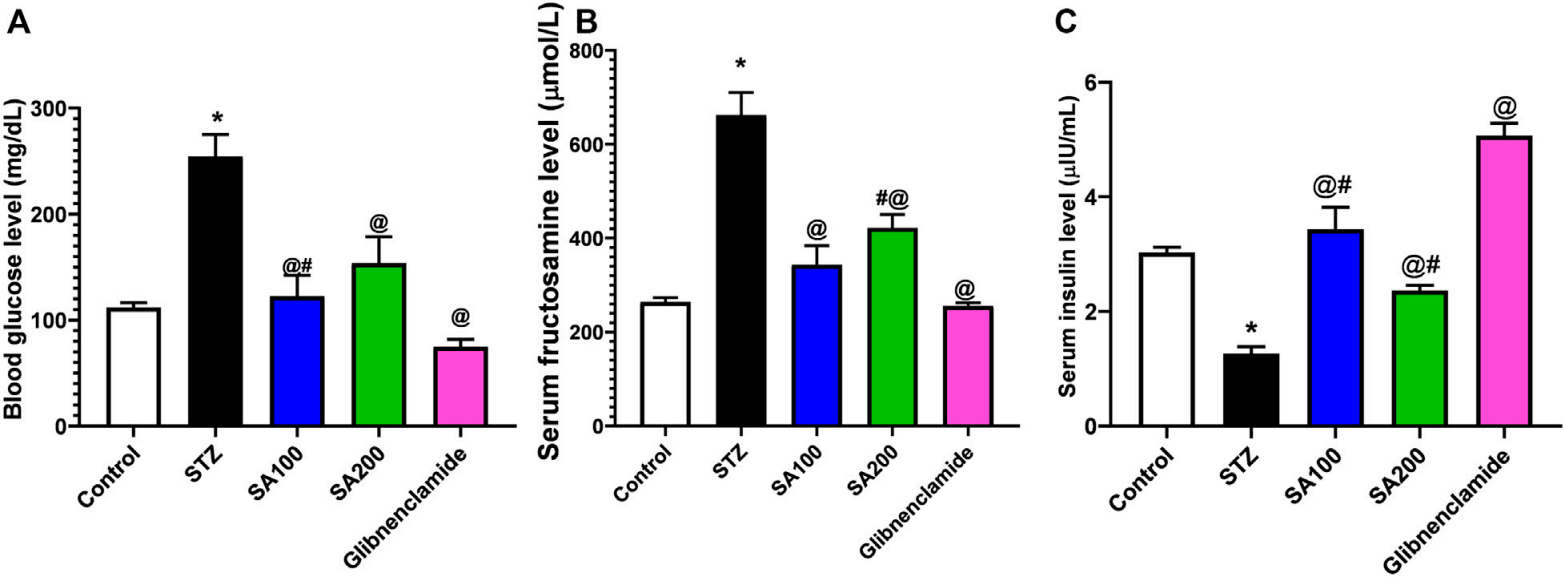
Results from the oral administration of the leaf extract of *S. aqueum* (SA) on the levels of **(A)** blood glucose **(B)** serum fructosamine **(C)** serum insulin in STZ-induced diabetic rats. Results are presented as mean ± SEM. *Significant difference related to the normal control group, ^@^related to STZ diabetic rats, ^#^related to reference drug group, glibenclamide, at *p <* 0.05 by One-Way ANOVA and Tukey’s *post hoc* test, n = 6.

Figure 2 describes the results from the oral administration of SA leaf extract (100 and 200 mg/kg) on the levels of blood glucose, fructosamine, and serum insulin in STZ-induced diabetic rats. Injection of STZ caused a significant increase in blood glucose and serum fructosamine and a significant reduction of serum insulin level compared to normal rats. Both SA and glibenclamide administration significantly (*p* < 0.05) decreased the elevated levels of blood glucose and fructosamine and increased the diminished serum insulin level compared to the STZ diabetic group. Glibenclamide produced better control of glycemic parameters than the extract. Noteworthy, SA (100 mg/kg) demonstrated better activities than SA (200 mg/kg).

### Effects of SA on the Structural Derangements of the Pancreas of STZ Animals

**FIGURE 3:**
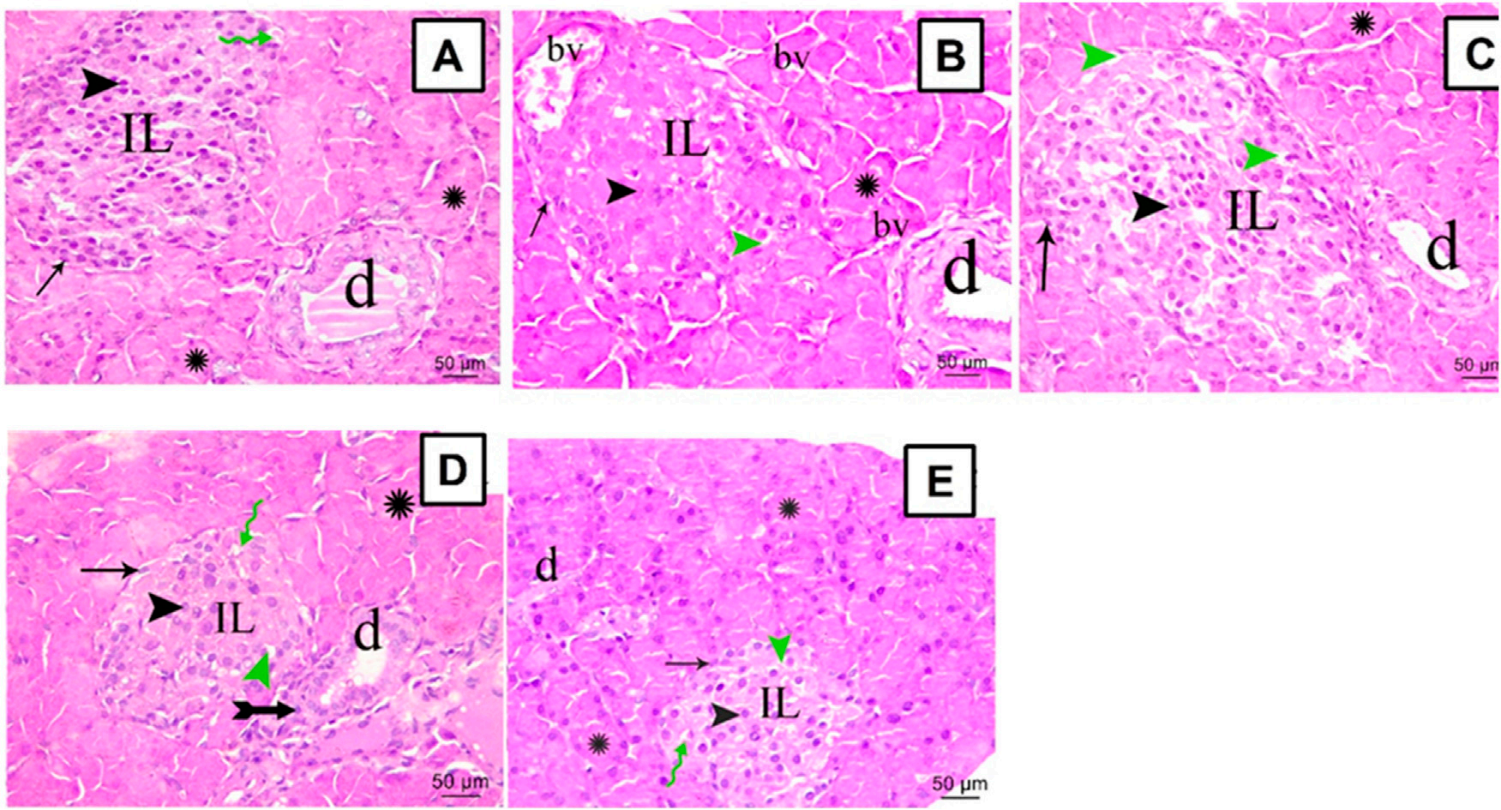
Stained photomicrographs with H&E of pancreatic sections from normal rats **(A)**, STZ diabetic rats **(B)**, *S. aqueum* (SA) extract (100 mg/kg) **(C)**, *S. aqueum* (SA) extract (200 mg/kg) **(D)**, and the reference drug, glibenclamide **(E)**. Green Zigzag arrow, arrow, arrowhead, green arrowhead, asterisk, and tailed arrow represent blood capillary, α cells, β cells, areas of vacuolation, acini, and inflammatory cells. (IL) Langerhans islets (bv) blood vessels and (d) duct. H and E; scale bar: 50 μm.

The sections from all groups were stained using H&E and their histopathological appearance was examined under the microscope, scale bar = 50 μm. Normal rats showed a common structure with closely packed acini lined by pyramidal cells with basal nuclei denoting the exocrine portion of the pancreas (Figure 3A). The Langerhans islets of STZ rats revealed substantial vacuolations within and between their secretory cells and seemed disturbed with an infrequent profile. Furthermore, marked congestion in multiple large blood vessels within Langerhans islets and between acini was observed (Figure 3B). Rats treated with SA100 restored their Langerhans islets but displayed, still, marked vacuolation within them (Figure 3C). On the other hand, rats treated with SA 200 or glibenclamide markedly restored pancreatic endocrinal portion; nearly normal morphology apart from very few inflammatory cells and areas of vacuolation (Figure 3D, E).

### Effects of SA on Collagen Deposition in Pancreas of STZ Animals

**FIGURE 4:**
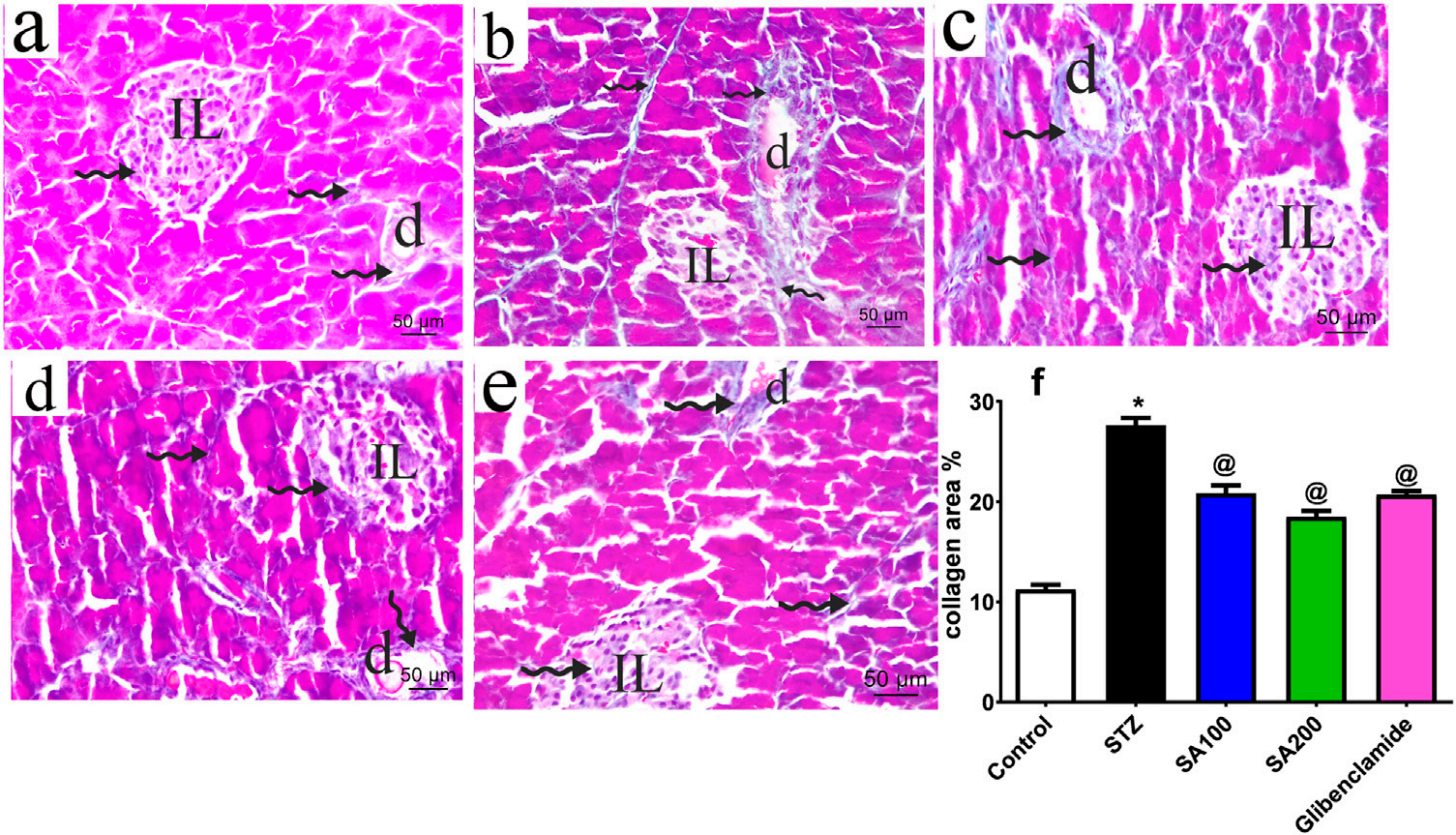
Stained photomicrographs with Mallory trichrome of pancreatic sections presenting collage fibers distribution from normal rats **(A)**, STZ diabetic rats **(B)**, *S. aqueum* (SA) extract (100 mg/kg) **(C)**, *S. aqueum* (SA) extract (200 mg/kg) **(D)**, the reference drug, glibenclamide **(E)**, and collagen area percent (%) **(F)**. Results are presented as mean ± SEM. *Significant difference related to the normal control group and ^@^related to STZ diabetic rats at *p <* 0.05 by One-Way ANOVA and Tukey *post hoc* test, n = 6. Zigzag arrow indicating blue staining of the collagen of (IL) Langerhans islets and **(D)** duct. Scale bar: 50 μm.

The pancreatic sections from all groups were stained using Mallory trichrome and the collagen fibers distribution was assessed under the microscope, scale bar = 50 μm. Normal rats showed normal collagen fibers distribution around pancreatic blood vessels, ducts, and acini with few scattered collagen fibers within Langerhans islets (Figure 4A). STZ rats displayed increased magnitude of collagen within Langerhans islets, around ducts and between the acini with a significant amount compared to the normal rats (Figure 4B). The two doses of the extract and glibenclamide groups downregulated collagen fibers (Figures 4C–E) and displayed a lower area% of collagen than STZ rats (*p* ˂ 0.05) (Figure 4F).

### Effects of SA on the Levels of Pancreatic MDA and GSH in STZ Animals

**FIGURE 5:**
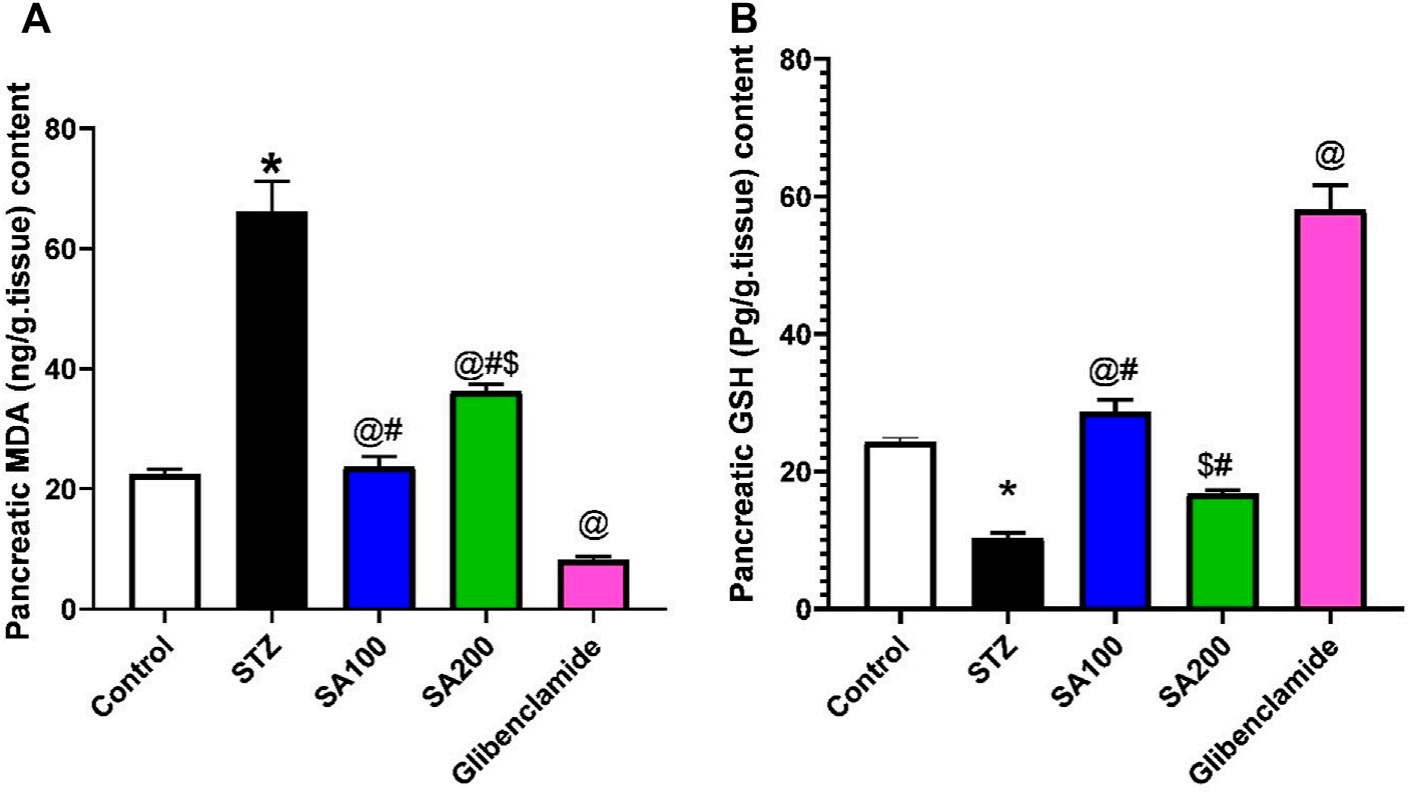
Results from the oral administration of the leaf extract of *S. aqueum* (SA) on the levels of pancreatic **(A)** malondialdehyde (MDA) and **(B)** reduced glutathione (GSH) in STZ diabetic animals. Results are presented as mean ± SEM. *Significant difference related to the normal control group, ^@^related to STZ diabetic rats, ^#^related to reference drug group, glibenclamide, and ^$^related to SA100 at *p <* 0.05 by One-Way ANOVA and Tukey *post hoc* test, n = 6.

We studied the effect of SA on pancreatic oxidative stress. STZ increased pancreatic MDA and decreased GSH contents compared to the normal rats. We found that SA oral administration significantly diminished the elevated levels of MDA and increased the declined levels of GSH compared to STZ rats. Noteworthy, SA (100 mg/kg) exhibited better activities than SA (200 mg/kg) in both parameters, Figure 5.

### Effects of SA on the Levels of Pancreatic Nrf-2 and HO-1 in STZ Animals

**FIGURE 6:**
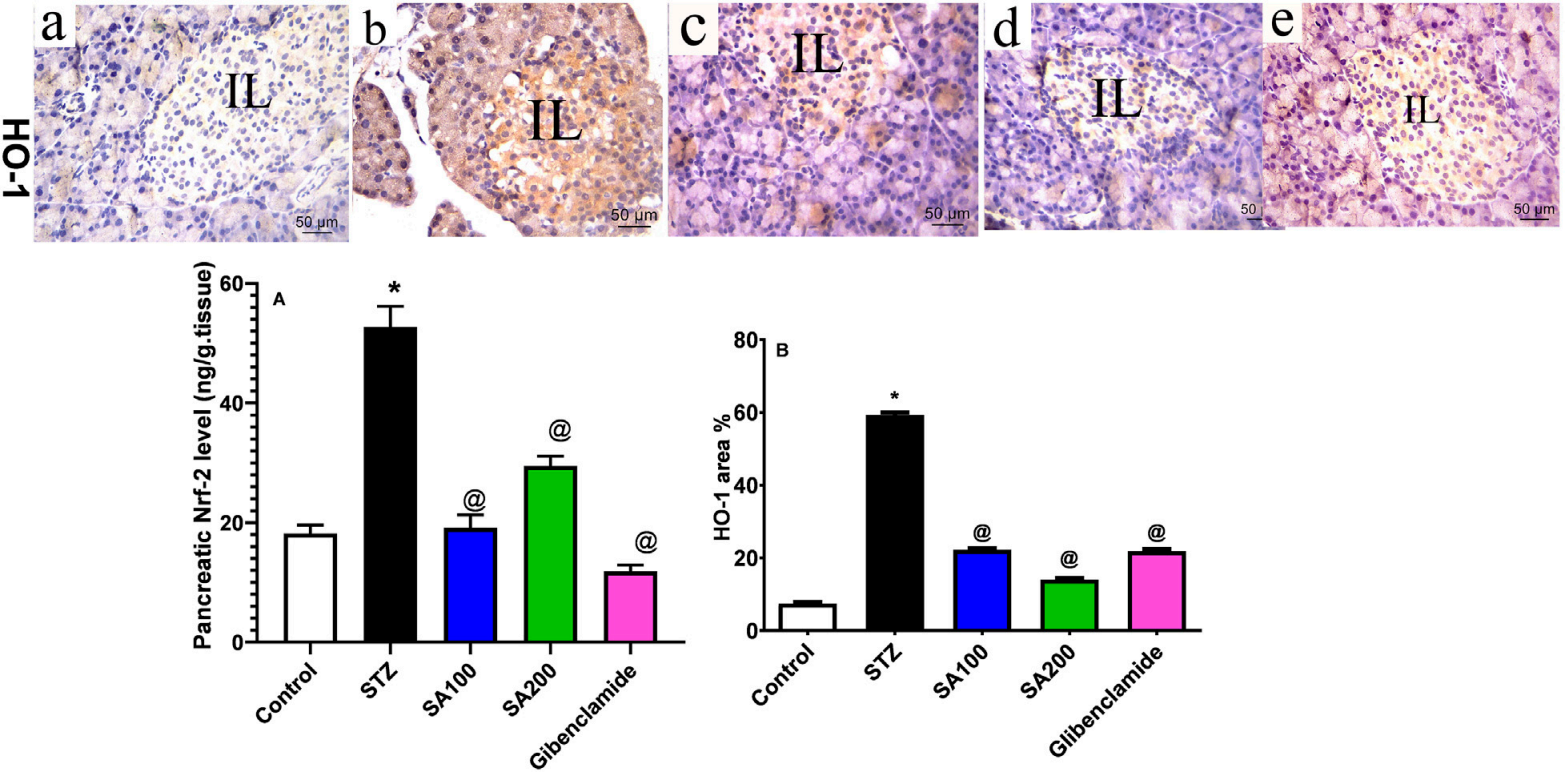
**(Upper panel)** Immunostained photomicrographs pancreatic sections presenting nuclear erythroid factor 2 (Nrf-2) and heme oxygenase-1 (HO-1). Normal rats **(A)**, STZ diabetic rats **(B)**, *S. aqueum* (SA) extract (100 mg/kg) **(C)**, *S. aqueum* (SA) extract (200 mg/kg) **(D)**, and the reference drug, glibenclamide **(E)**. **(Lower panel)** Nrf-2 area percentage (%) **(A)**. HO-1 area percentage (%) **(B)**. Results are presented as mean ± SEM. *Significant difference related to the normal control group and ^@^related to STZ diabetic rats at *p <* 0.05 by One-Way ANOVA and Tukey’s *post hoc* test, n = 6.

STZ injection drastically elevated Nrf-2 and HO-1 by 2.9 and 8 folds, respectively, compared to the normal animals. Both tested doses significantly declined the elevated levels of Nrf-2 by 2.8- and 1.8-fold, respectively, vs. 4.4-fold for glibenclamide; however, SA (100 mg/kg) displayed better activities than SA (200 mg/kg) Figure 6A. On the other hand, SA oral administration significantly reduced the elevated levels of HO-1 in a dose-dependent manner using SA (100 mg/kg) and SA (200 mg/kg) (by 2.7- and 4.2-fold, respectively, vs. 2.7-fold for glibenclamide), Figure 6B. Interestingly, no significant difference was observed between the two tested doses and the reference drug, glibenclamide, Figure 6B.

### Effects of SA on the Levels of Pancreatic TNF-α and TLR-4 in STZ Animals

**FIGURE 7:**
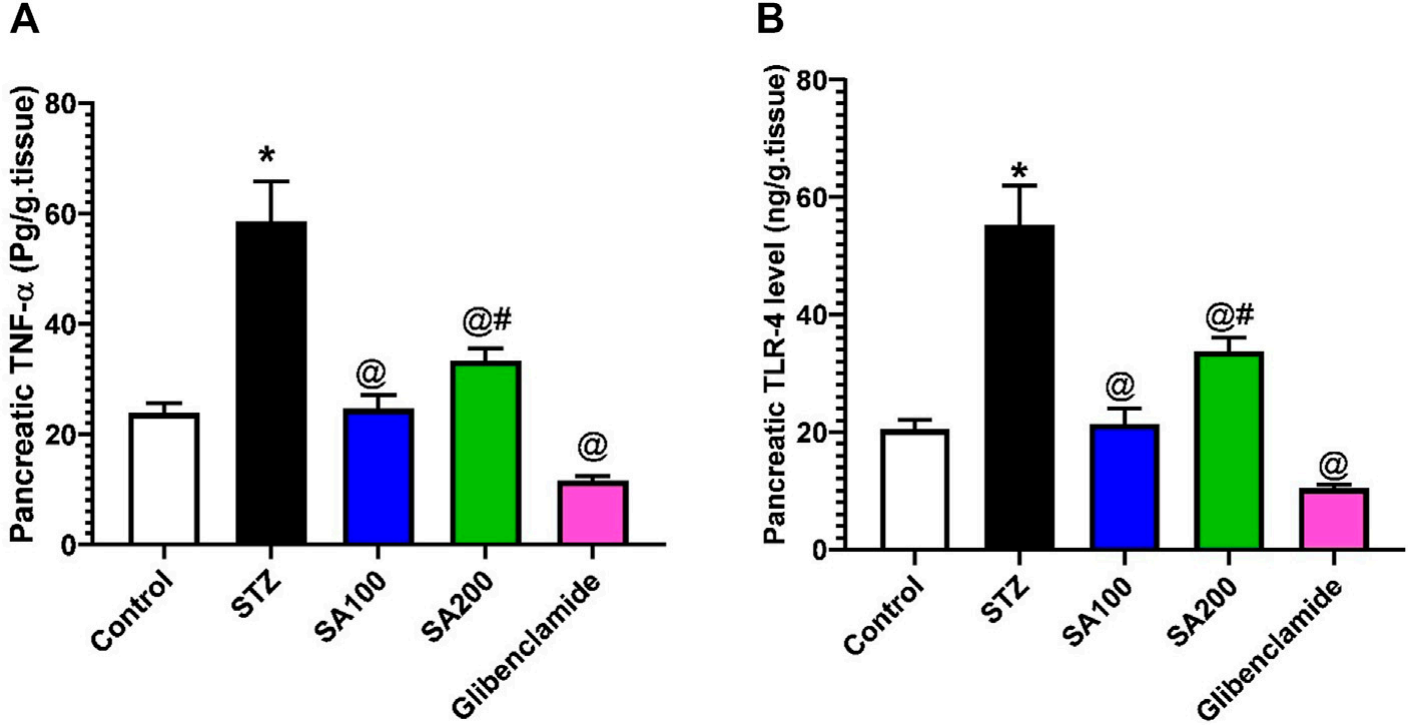
Results from the oral administration of the leaf extract of *S. aqueum* (SA) on the levels of pancreatic **(A)** tumor necrosis factor-alpha (TNF-α) and **(B)** toll-like receptor 4 (TLR-4) in STZ diabetic animals. Results are presented as mean ± SEM. *Significant difference related to normal control group, ^@^related to STZ diabetic rats, and ^#^related to reference drug group, glibenclamide at *p <* 0.05 by One-Way ANOVA and Tukey’s *post hoc* test, n = 6.

We also investigated the effect of STZ on the local inflammation of pancreatic tissues. Our results revealed that STZ injection drastically elevated the levels of TNF-α and TLR-4 related to the normal animals. Oral administration of SA or glibenclamide significantly declined the elevated levels; however, SA (100 mg/kg) exhibited better activities than SA (200 mg/kg), as shown in Figure 7.

We also utilized the *in silico* molecular docking tool to investigate the potential of the major SA extract components to bind and hence interfere with the activation of TLR4. The X-ray structure of the TLR4-MD2 complex demonstrating the binding pocket of LPS was downloaded from pdb and used in this study. In total, 62 compounds belonging to phenolic acids, flavonoids, glycosides, and tannins were docked into the binding site of LPS on the TLR4-MD2.

As shown in Table 1, 11 compounds showed strong binding to the target protein with binding free energy less than −15 kcal/mol. Only eight compounds were not able to fit into the binding site and showed high binding energy (more than −10 kcal/mol). The rest of the compounds showed appropriate binding energy ranging between −10 and −15 kcal/mol. The docked compounds were able to iterate some of the reported interactions between the complex protein and LPS, such as the interactions with Phe119, Ser120, Phe151, and Lys263. In addition, our compounds furnished some extra stabilizing interactions with some more amino acids in the binding site, such as Asn293, Arg337, and Gln339. These results showcase the strong potential of the extract components to target the TLR4 and represent some lead compounds that could be utilized as a base for designing some novel TLR4 inhibitors. The best four compounds were theaflavin 3′-*O*-gallate (S = −19.32 kcal/mol), samarangenin A (S = −18.17 kcal/mol), castalagin (S = −17.46 kcal/mol), and galloylquinic acid (S = −16.84 kcal/mol).

**TABLE 1:** Docking scores of *S. aqueum* compounds into the active site of TLR4 using *in silico* molecular docking experiments.

Compounds^a^	Score function (Kcal/mol)
Quinic acid	−9.25
Caffeic acid	−9.79
Caffeoyl hexoside	−9.00
Digalloyl hexose	−14.54
Ellagic acid	−10.02
Feruloylquinic acid	−11.77
Galloyl hexose	−12.60
Galloylquinic acid	−16.84
*p*-Coumaric acid hexoside	−8.52
*p*-Coumaroylquinic acid	−8.72
Cryptostrobin	−7.87
Europetin	−10.63
Europetin rhamnoside	−12.44
Isorhamnetin	−10.32
Isorhamnetin 3-*O*-glucoside	−10.49
Isorhamnetin 3-*O*-glucoside 7-*O*-rhamnoside	−15.73
Isorhamnetin 3-*O*-rutinoside	−12.46
Isorhamntin pentoside	−11.56
Ligstroside	−10.10
Myricetin	−10.68
Myricetin 3-*O*-arabinoside	−15.75
Myricetin 3-*O*-galactoside	−14.40
Myricetin 3-*O*-rutinoside	−14.80
Myricetin galloly-hexoside	−16.05
Myricetin3-*O*-rhamnoside	−13.14
Myrigalone B	−10.16
Myrigalone G	−9.56
Quercetin 3,4′-*O*-diglucoside	−13.69
Quercetin 3-*O*-(6″-acetyl-galactoside) 7-O-rhamnoside	−13.86
Quercetin 3-*O*-arabinoside	−14.84
Quercetin 3-*O*-galactoside	−16.22
Quercetin 3-*O*-galactoside 7-O-rhamnoside	−13.96
Quercetin 3-*O*-rutinoside	−11.27
Quercetin 3-*O*-sophoroside	−13.38
Quercetin 3-*O*-xyloside	−11.95
Quercetin 4′-*O*-glucoside	−12.94
Quercetin 3-*O*-rhamnoside	−13.37
Tricin	−11.85
(-)-Epicatechin	−11.33
(-)-Epicatechin gallate	−10.89
(-)-Epigallocatechin	−11.44
(-)-epigallocatechin-3-*O*-gallate	−13.67
(+)-Catechin	−10.69
(+)-Catechin 3-*O*-gallate	−12.45
(+)-Gallocatechin	−12.08
Epiafzelechin	−9.38
Castalagin	−17.46
Casuarinin	−16.62
Catechin 3′-glucoside	−13.01
Epiafzelechin-(4b->8)-epicatechin 3,3′-digallate	−14.09
Gallocatechin-(4alpha->6)-catechin	−15.49
Pedunculagin	−14.78
Procyanidin C1	−14.63
Procyanidin dimer B1	−14.62
Procyanidin dimer B2	−13.46
Procyanidin dimer B4	−12.86
Procyanidin dimer B5	−12.12
Procyanidin dimer B7	−13.97
Samarangenin A	−18.17
Tellimagrandin II	−15.48
Theaflavin	−13.36
Theaflavin 3′-*O*-gallate	−19.32

a Previously characterized in Sobeh et al., 2018.

### Effects of SA on the Levels of Pancreatic TRAF6 and MyD88 in STZ Animals

**FIGURE 8:**
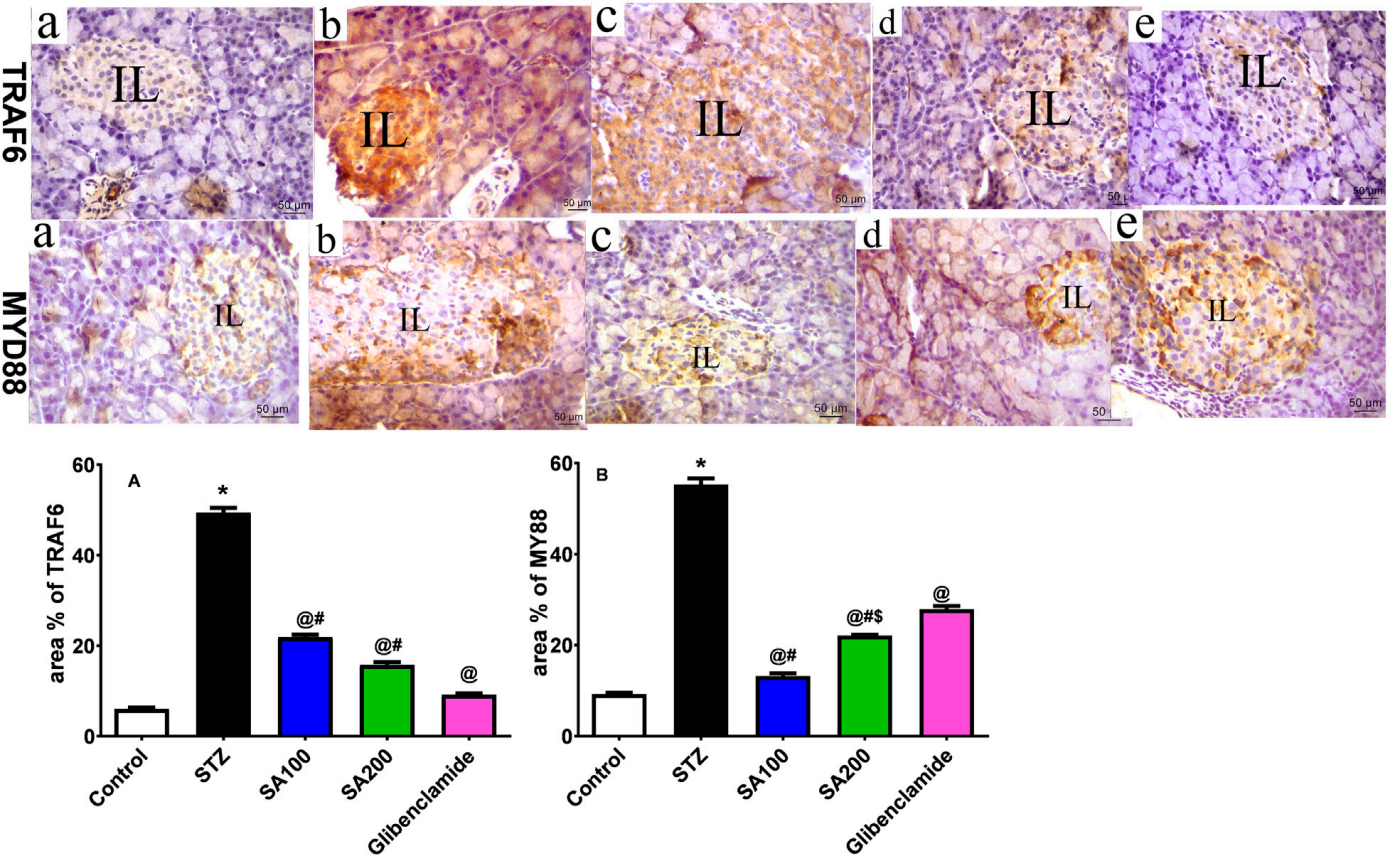
**(Upper panel)** Immunostained photomicrographs of pancreatic sections presenting tumor necrosis factor receptor (TNFR)-associated factor 6 (TRAF6) and myeloid differentiation factor 88 (MyD88). Normal rats **(A)**, STZ diabetic rats **(B)**, *S. aqueum* (SA) extract (100 mg/kg) **(C)**, *S. aqueum* (SA) extract (200 mg/kg) **(D)**, and the reference drug, glibenclamide **(E)**. **(lower panel)** TRAF6 area percentage (%) **(A)**. MyD88 area percentage (%) **(B)**. Results are presented as mean ± SEM. *Significant difference related to the normal control group, ^@^related to STZ diabetic rats, ^#^related to reference drug group, glibenclamide, and ^$^related to SA100 at *p <* 0.05 by One-Way ANOVA and Tukey’s *post hoc* test, n = 6.

STZ injection drastically elevated TRAF6 and MyD88 by 8.2-fold and 5.9-fold, respectively, compared to the normal animals. SA oral administration significantly reduced the elevated levels of TRAF6 by 2.3- and 3.1-fold using SA (100 mg/kg) and SA (200 mg/kg), respectively, and 5.4-fold for glibenclamide, as shown in Figure 8A. Both doses significantly diminished the elevated levels of MyD88; however, SA (100 mg/kg) displayed better activities than SA (200 mg/kg). Noteworthy, both doses expressed better activities on MYD88 than the reference drug, glibenclamide, as shown in Figure 8B.

### Effects of SA on the Levels of Liver IRS-2, p-AKT, and GLUT4 in STZ Animals

**FIGURE 9:**
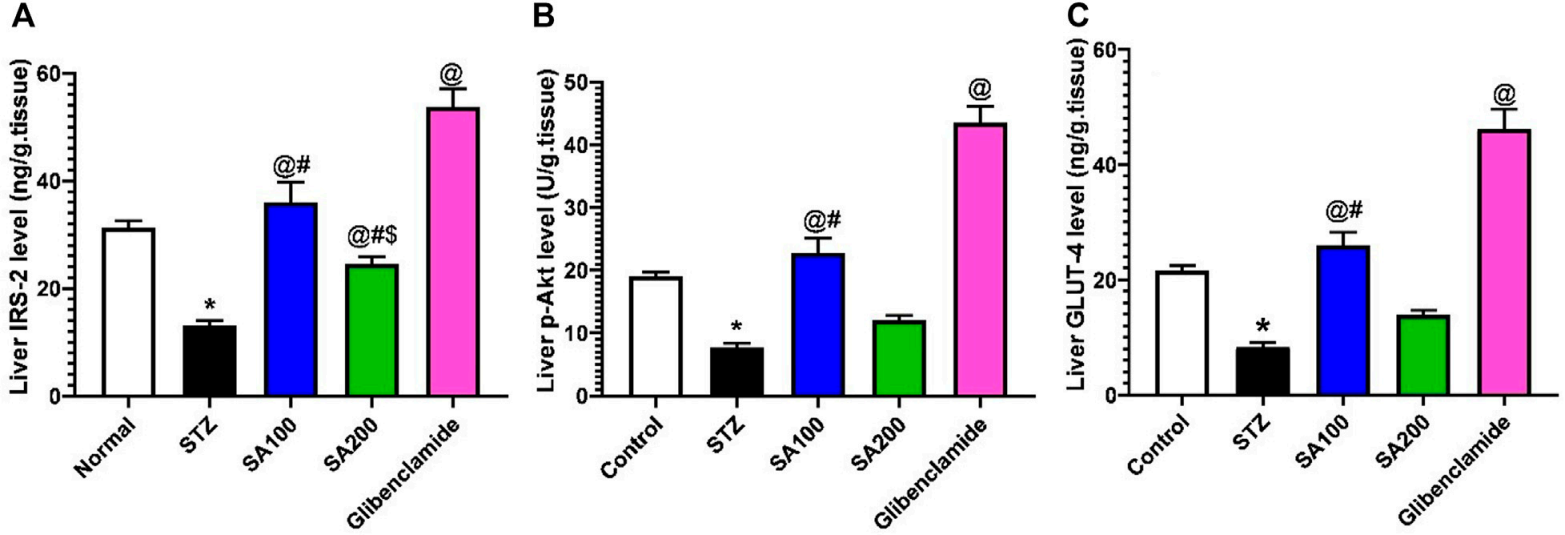
Results from the oral administration of the leaf extract of *S. aqueum* (SA) on the levels of liver **(A)** insulin receptor substrate-2 (IRS-2), **(B)** phosphorylated protein kinase B (p-AKT), and **(C)** glucose transporter 4 (GLUT4) in STZ diabetic animals. Results are presented as mean ± SEM. *Significant difference related to the normal control group, ^@^related to STZ diabetic rats, ^#^related to the reference drug group, glibenclamide, and ^$^related to SA 100 at *p <* 0.05 by One-Way ANOVA and Tukey’s *post hoc* test, n = 6.

Injection of STZ drastically declined the levels of IRS-2, p-AKT, and GLUT4 by 2.4-, 2.5-, and 2.6-fold, respectively, in the rats’ hepatic tissue when compared to the normal control group, *p* < 0.05. SA (100 mg/kg) and glibenclamide administration scientifically increased the declined levels related to the STZ diabetic group. However, SA (200 mg/kg) significantly increased the IRS-2 level only but not p-AKT nor GLUT4, as shown in Figure 9.

## Discussion

We investigated the possible antidiabetic effects of the leaf extract of *S. aqueum* in a rat model of type 2 diabetes compared to glibenclamide, a well-known oral hypoglycemic drug used to treat this type of diabetes*.* The current results indicate that SA extract possesses antidiabetic effects as it decreases blood glucose level and serum fructosamine level and elevates serum insulin level*.* Blood glucose enables diagnosis, monitoring the management, and predicting the vascular complications of the disease (Krentz and Hompesch 2016). Fructosamine, also known as glycated serum protein, is formed upon combining glucose with proteins such as albumin and other serum proteins in the glycosylation process. Since serum proteins are present in the blood for about 21 days, fructosamine level can reflect the glucose level over almost 3 weeks (Link and JacquieRand 2008). The reduction of both blood glucose and serum fructosamine levels may be attributed to the increased insulin secretion by the extract observed in this study. Insulin is considered among the key regulators of glucose homeostasis as it helps move glucose from the bloodstream to the body cells, where it can be utilized for energy generation. The majority of oral hypoglycemic drugs, such as glibenclamide used to manage type 2 diabetes, work by stimulating the secretion of insulin by the pancreatic beta cells, hence leading to a rise in the serum insulin level following administration (Ganesan and Sultan 2018). The increased insulin secretion by the extract may be attributed to the improvement of structural derangements of pancreatic islets induced by STZ administration. Furthermore, it may also be related to the suppression of collagen fibers deposition observed in STZ-treated rats (Mahmoud et al., 2021).

We investigated the possible underlying mechanisms of the extract in improving both diabetic status and structural changes of the pancreas. We found that the extract suppressed both pancreatic oxidative stress and inflammation. Oxidative stress is a key player in the development of both cardiovascular and microvascular complications of diabetes disease. It is well reported that STZ injection is accompanied by prolonged and excessive ROS production in animals. The elevated glucose level leads to excessive production of pyruvate in cells that is subject to oxidation in the citric acid cycle, leading to an overflux of the electron donors FADH2 and NADH towards the electron transport chain. This causes a blockage in the electron transfer when a critical threshold of voltage gradient is reached across the mitochondrial membrane. As a result, electrons back up and get donated to molecular O_2_ generating superoxide, which is converted in turn to more reactive oxygen species (ROS) that cause tissue damage in various ways (Wallace 1992). From another perspective, high intracellular glucose concentrations increase the flux of the polyol pathway, in which the aldose reductase enzyme changes glucose into sorbitol utilizing NADPH as a cofactor leading to its depletion. NADPH is also an important cofactor for generating the crucial ROS scavenger and reducing glutathione (GSH). Thus, depletion of NADPH could exacerbate the status of intracellular oxidative stress (Chung et al., 2003). Our results revealed that in STZ animals, SA extract ameliorated the enhanced ROS production where it reduced the level of pancreatic MDA, a lipid peroxidation product, and increased the level of pancreatic GSH, an endogenous antioxidant. It is noteworthy to mention that antidiabetic remedies with antioxidant potential are of great value in managing type 2 diabetes and minimizing its vascular complications.

Nuclear erythroid factor-2 (Nrf-2) is a critical factor in regulating the intracellular antioxidant defense mechanisms. It is well proved that Nrf2 is stimulated during conditions of elevated oxidative stress, such as chemical stress and hypoxia (Nguyen et al., 2009). Previous studies have demonstrated the overexpression of Nrf2 during a renal ischemic-reperfusion injury in mice models and its protective role in mitigating such renal injury and inflammation in the experimental animals and hemodialysis patients (Pedruzzi et al., 2015; Shokeir et al., 2015). Moreover, Nrf2 regulates the expression of heme oxygenase-1 (HO-1), which plays important antioxidant, anti-inflammatory, and antiproliferative roles in vascular cells (Abraham and Kappas 2008; J. E.; Kim et al., 2019). A study by Kim et al. showcased the relationship between Nrf2 and HO-1, where it was demonstrated that inhibition of Nrf2 in tubular cells blocked HO-1 activation following iohexol-induced renal injury (J. E. Kim et al., 2019). Given these findings, it is clear that oxidative stress conditions contribute to a marked rise in the intracellular levels of Nrf2 and HO-1. We observed in our study a significant increase in the levels of Nrf2 and HO-1 in the pancreatic tissues of STZ diabetic rats. This effect could be attenuated by the administration of some molecules or remedies with profound antioxidant potential. As expected, SA extract diminished the elevation of both Nrf2 and HO-1, indicating potent antioxidant effects.

Another possible mechanism of the antidiabetic effect of the extract is the suppression of pancreatic inflammation. Recent studies reported the critical role of some specific cytokines, chemokines, and proteins associated with inflammation and oxidative stress in the pathogenesis of type 2 diabetes, opening the way towards novel remedies for managing the disease (Mahmoud et al., 2021). For instance, increased expression of TNFα was observed in the pancreatic cells of diabetic animals (Ladefoged and Buschard 2013; Mahmoud et al., 2021). In addition, the toll-like receptor (TLR) family could have a critical role in the inflammation associated with diabetes disease. This family comprises ten transmembrane proteins engaged in the innate immunity against various pathogens and infections. These receptor members are expressed on either the cell surface or the endosomes and can recognize different types of molecules expressed by pathogens, including lipopolysaccharides, peptidoglycan, and flagellin, after both innate immune and inflammatory responses are mediated (Netea et al., 2004).

The TLR-4 member of this family is reported to be among the key factors involved in inducing a pro-inflammatory response following infectious or noninfectious stimuli. It is known as the sensing receptor for the lipopolysaccharides of Gram-negative bacteria and is able to bind specific endogenous molecules resulting upon different tissue injuries (Molteni et al., 2016). TLR4 is activated upon the binding of lipopolysaccharide (LPS) molecule; however, this cannot be possible without the involvement of the cluster of differentiation 14 (CD14) and the adapter molecule MD-2 that mediate the signal activation cascade. It is demonstrated that TLR4-MD2 dimer must be formed first to facilitate the binding of the LPS to the MD-2, leading to the activation of TLR4, resulting in the production of various pro-inflammatory interferons and cytokines (S. J. Kim and HoKim 2017). Recently, TLR4 has grabbed much attention due to its role in the progression of diabetes type 2, insulin resistance, in addition to its profound contribution in damaging beta cells in the pancreas (Ladefoged and Buschard 2013). In this study, STZ injection increased the expression of TLR4 and its adaptor protein, MYD88. This adaptor protein is involved in the activation of inflammatory pathways and found to be involved in the prognosis of diabetes, where its deficiency has offered considerable protection against STZ-induced diabetes in mice (Androulidaki et al., 2018). STZ also activated the TLR-4 signaling pathway, as revealed by increasing the level of TRAF 6, which is an important signal transducer in TLR4 and NF-κB pro-inflammatory signaling pathway (Yoshida et al., 2004; Cohen 2014). It was recently reported that TRAF6 was able to mediate endothelial dysfunction induced by a high glucose level through NF-κB- and AP-1-dependent signaling. Knocking down TRAF6 showed to inhibit the degradation of IκB-α and JNK phosphorylation that are induced by a high glucose level; thus, targeting TRAF6 could be of value to delay the progression of vascular diseases associated with diabetes (R. Liu et al., 2018). Herein, the extract not only suppressed the pancreatic expression of TLR-4 and its adaptor protein, MYD88, but also decreased TRAF6 and TNF-α production in pancreatic tissues indicating potent anti-inflammatory effects, explaining the improvement of pancreatic structural changes caused by the extract. The *in vivo* effects of the extract on TLR-4 were also confirmed by an *in silico* study, which showed strong binding of the extract's active constituents with this receptor.

Diabetes type 2 is usually preceded by the development of insulin resistance in the target tissues. Currently, the family of the insulin receptor substrate (IRS) proteins is identified among the molecular causes beyond the initiation and progression of insulin resistance. Phosphorylation of IRS proteins by the insulin receptor activates a complex of secondary messenger cascades regulating glucose and lipids metabolism. It was reported that ablation of IRS-2 in mice leads to many type 2 diabetes hallmarks, including peripheral insulin resistance conjoined with a lack of compensatory beta cells expansion, ending up with hyperglycemia and diabetes, and followed by premature death (Withers et al., 1998; Kubota et al., 2000). The peripheral uptake of glucose to the skeletal muscles, which represent the major disposal site for glucose, is promoted by the insulin-dependent recruitment and activation of Akt/PKB (also known as p-AKT), a serine/threonine protein kinase. Phosphorylation and hence activation of p-AKT by further kinases leads to translocation of GLUTs to the cell membrane to facilitate glucose uptake (Lu et al., 2012). The essential glucose transporter, liver GLUT4, is among those ruled by the signaling pathway p-AKT. In the current study, we observed that both doses of the extract produced comparable effects to glibenclamide on the structural changes of the pancreas; however, the low dose of the SA extract produced better effects than the high dose on the glycemic parameters. In an attempt to explain this difference between the two dose levels, we investigated the effects of the extract on the hepatic insulin signaling pathway. We found that both dose levels of SA extract increased IRS-2, but only the low dose increased phosphorylated protein kinase B (AK) and hepatic GLUT-4 levels, which is responsible for glucose uptake. These findings could explain the better effects of the low dose over the high dose on glycemic parameters.

The obtained pharmacological activities might be associated with the existence of diverse classes of secondary metabolites that bind with several molecular targets (Salaj et al., 2021). For instance, myricitrin, a major compound at the extract, significantly reduced the levels of blood glucose and TLR-4 and increased the levels of plasma insulin and TNF-α compared to the diabetic mice (D. Y. Kim et al., 2020).

The green tea polyphenol, EGCG (among the major compounds of the extract), significantly reduced the lipid profile and oxidative stress and ameliorated glycemic control and insulin sensitivity in the type 2 diabetes rat model (Li et al., 2020). SA contained 11 quercetin glucosides and galloylated derivatives; they are renowned for their antidiabetic effects (Sobeh et al., 2018; Salehi et al., 2020; Tan et al., 2021). Moreover, the two flavonoids, EGGC and quercetin, are commonly found in edible plants. A recent study highlighted synergistic protective effects of quercetin and EGCG against pancreatic cell damage in which they enhanced glucose-stimulated insulin secretion and reversed STZ-induced cells damage (H. Liu et al., 2021). Two isorhamnetin derivatives were also annotated in the extract. A recent work concluded that isorhamnetin could be utilized to ameliorate insulin resistance related to type 2 diabetes (Matboli et al., 2021).

SA is rich in tannins (35 compounds); it is well-established that tannins exert substantial antidiabetic activities (Rauf et al., 2019; Blahova et al., 2021). Several tannins-rich extracts furnished similar activities, such as *Ximenia americana* var*. caffra* and *Albizia harveyi* (Sobeh et al., 2017a; 2017b). Of note, several plant species from the Myrtaceae family displayed pronounced antidiabetic activities in rats. These include *Eugenia uniflora* and *Syzygium jambos* (Sobeh et al., 2019; Mahmoud et al., 2021).

## Conclusion

In this work, we highlighted the protective effects of a leaf extract from *S. aqueum* against STZ-induced diabetes in rats. The extract ameliorated the deleterious effects of ROS, increased insulin secretion, reduced TLR-4, MYD88, pro-inflammatory cytokine, TNF-α, and TRAF-6 levels in pancreatic tissues, restored Langerhans islets morphology, and reduced the disposition of collagen. To sum up, the observed biological activities extend the traditional use and reflect the therapeutic potential of *S. aqueum* in treating diabetes. Further detailed experiments are needed before translating these activities into applications in humans.

## Data Availability

The original contributions presented in the study are included in the article/Supplementary Material; further inquiries can be directed to the corresponding authors.
